# Direct Air Capture of CO_2_: Mechanism-Guided Materials, Process Integration, and Scalable Carbon Removal

**DOI:** 10.3390/molecules31173056

**Published:** 2026-08-31

**Authors:** Xinyi Wei, S. K. Kot-Cheung, Jing Sun

**Affiliations:** 1 North Alabama International College of Engineering and Technology, Guizhou University, Guiyang 550001, China; 18533603988@163.com; 2 College of Science, Northeast Forestry University, Harbin 150040, China; 3 College of Science, Heilongjiang Institute of Technology, Harbin 150050, China

**Keywords:** direct air capture, carbon dioxide removal, CO_2_ sorbents, structured sorbents, net carbon removal

## Abstract

Direct air capture of CO_2_ (DAC) is an emerging engineered carbon removal technology designed to extract CO_2_ directly from ambient air and support long-term net-negative emission pathways. Unlike point-source carbon capture, DAC operates under ultradilute CO_2_ conditions, where the low partial pressure of CO_2_, large air-processing demand, humidity fluctuations, and regeneration energy requirements impose coupled thermodynamic, kinetic, and engineering constraints. While numerous reviews have addressed DAC materials or specific process configurations, a systematic account of how capture performance is progressively lost through the transition from molecular binding to material shaping, contactor operation, regeneration, and final storage remains lacking. This review fills this gap by adopting a performance-transfer framework that bridges capture chemistry, sorbent architecture, contactor engineering, and scalable deployment. We systematically survey the literature of the past decade across capture chemistries, sorbent design principles, structured contactors, regeneration strategies, and system integration, with a focus on studies that report cyclic working capacity, regeneration energy, and material stability under realistic conditions. Rather than enumerating material properties, we analyze the cascading losses introduced at each scale and identify the key bottlenecks limiting net carbon removal. Based on this analysis, we propose a staged roadmap from standardized material reporting to integrated DAC with carbon storage, aiming to guide future research toward verifiable and durable CO_2_ removal.

## 1. Introduction

Direct air capture of carbon dioxide extracts CO_2_ from ambient air and is designed to support net-negative emission pathways [1]. Emission reduction alone cannot fully eliminate residual emissions from hard-to-abate sectors or reduce the atmospheric CO_2_ stock that has already accumulated [2]. Conventional carbon capture is aimed at concentrated point sources such as coal-fired plants, cement kilns, and steel mills, and its primary effect is to reduce new emissions [3,4]. DAC differs from point-source capture in both its feed composition and its climate function [5]. When DAC is coupled with geological storage, mineral carbonation, or other durable sinks, it delivers engineered negative emissions [6,7]. This technology should therefore be regarded as a distinct carbon-removal pathway for residual, dispersed, and mobile sources rather than as a direct extension of point-source capture [8].

DAC offers considerable siting flexibility because atmospheric CO_2_ is distributed globally, and systems can be located near low-carbon energy sources, geological storage reservoirs, mineralization feedstocks, or existing infrastructure [9]. The climate benefit of DAC depends entirely on the fate of the captured carbon. Geological storage and mineral carbonation can provide durable removal when storage integrity and life-cycle emissions are properly verified. Concrete curing and long-lived carbonate products occupy an intermediate category whose climate value is contingent on retention time and accounting rules. By contrast, applications such as fuel synthesis, short-lived chemicals, greenhouse enrichment, microalgae cultivation, and indoor air management mainly represent carbon cycling, utilization, or local service rather than durable net removal. The climate value of DAC therefore hinges on whether the captured CO_2_ remains net-negative and long-lived after accounting for energy use, material footprint, process emissions, and monitoring requirements.

DAC faces a significantly higher separation barrier than point-source capture [10,11]. Ambient air contains approximately 400 ppm CO_2_, which is two to three orders of magnitude lower than typical flue gas concentrations [12]. This ultradilute condition weakens the thermodynamic driving force for adsorption or absorption and thus necessitates sufficient CO_2_ affinity in the sorbent or solvent. Excessive binding, however, raises regeneration energy and slows desorption, giving rise to the central trade-off in DAC chemistry: low-pressure capture requires strong affinity while low-energy regeneration demands reversibility [13]. Kinetic limitations are equally critical because at 400 ppm only a small number of CO_2_ molecules reach reactive sites per unit time. Equilibrium uptake measured after long exposure can overstate performance under finite residence time, so a viable capture mechanism must sustain uptake under low CO_2_ flux and wet air while remaining reversible within a practical cycle time [14,15].

Current DAC routes differ less by whether they bind CO_2_ than by where they pay the separation penalty. Liquid alkaline systems provide strong absorption and mature carbonate chemistry, but their viability at scale is governed by calcination heat, caustic recovery, water balance, and CO_2_ purification [15,16,17]. Solid amine sorbents lower regeneration temperatures, yet their advantage diminishes when amine sites become inaccessible, water uptake increases heat duty, oxidation shortens lifetime, or shaping raises pressure drop [18,19,20]. Moisture-swing systems reduce direct heat input but replace it with demands for rapid dry–wet cycling, controlled water activity, and sufficient CO_2_ product concentration [21,22]. Electrochemical DAC shifts regeneration toward electricity, but voltage loss, oxygen side reactions, membrane resistance, and electrode aging determine whether lower heat demand translates into lower net removal energy [23,24].

Although several recent reviews have summarized advances in DAC materials, regeneration strategies, process engineering and deployment challenges, most existing discussions have been organized around individual technology categories or specific research scales [1,25,26,27,28]. For example, materials-focused reviews have mainly compared sorbent chemistry, structural modification and adsorption performance, whereas process-oriented reviews have emphasized energy integration, equipment design and system optimization. However, the connection between these scales remains insufficiently discussed, particularly regarding how molecular-level CO_2_ binding advantages are progressively retained or lost during shaping, air contacting, regeneration and final carbon storage. This separation between material evaluation and system-level performance assessment makes it difficult to identify which material improvements can ultimately contribute to durable carbon removal.

To address this gap, this review does not aim to provide another classification of DAC materials or process configurations. Instead, it develops a performance-transfer perspective that follows the evolution of CO_2_ capture performance across different scales, from molecular interactions and sorbent architectures to structured contactors, regeneration processes and carbon fate. By analyzing the losses introduced during each transition, this review identifies the factors that determine whether laboratory-scale capture advantages can be converted into practical and verifiable carbon removal. The expected contribution of this work is therefore to provide a unified framework for understanding DAC performance limitations and to guide the development of scalable DAC systems with improved energy efficiency, durability and carbon-removal reliability.

This review adopts a performance-transfer framework that connects capture chemistry, sorbent architecture, contactor operation, regeneration, and carbon fate. Within this framework, molecular binding must translate into cyclic CO_2_ recovery, recovered CO_2_ must become a usable product stream, and that stream must be directed toward durable storage. Utilization and biological fixation pathways are discussed only where they affect carbon accounting, while the main focus remains engineered DAC for net removal and long-term CO_2_ storage.

The literature covered in this review was identified through searches in Web of Science and Scopus, supplemented by Google Scholar and reference tracking from relevant review articles and highly cited studies. The search emphasized publications since 2010 to capture the rapid development of DAC materials, processes, and deployment strategies while retaining earlier studies that established fundamental mechanisms. The search terms included combinations of direct air capture, carbon dioxide removal, CO_2_ sorbents, amine adsorption, hydroxide absorption, moisture swing, electrochemical capture, structured contactors, regeneration, and process integration. Studies were selected based on their relevance to practical DAC performance, with particular attention to cyclic working capacity, regeneration energy consumption, material stability under humid and oxidative conditions, and system-level performance evaluation. Reports based solely on equilibrium uptake without considering cyclic operation or practical constraints were interpreted with caution. The identified literature was analyzed according to five interconnected themes, including capture chemistry, sorbent design principles, contactor engineering, regeneration strategies, and system integration with carbon storage. This organization follows the performance-transfer framework proposed in this review and enables analysis of how CO_2_ capture advantages are maintained or diminished across different scales. Given the broad scope of DAC research and the diversity of experimental and modeling conditions, this review does not aim to provide a quantitative meta-analysis but instead synthesizes the dominant mechanisms, trade-offs, and bottlenecks reported across the literature.

**Figure 1:**
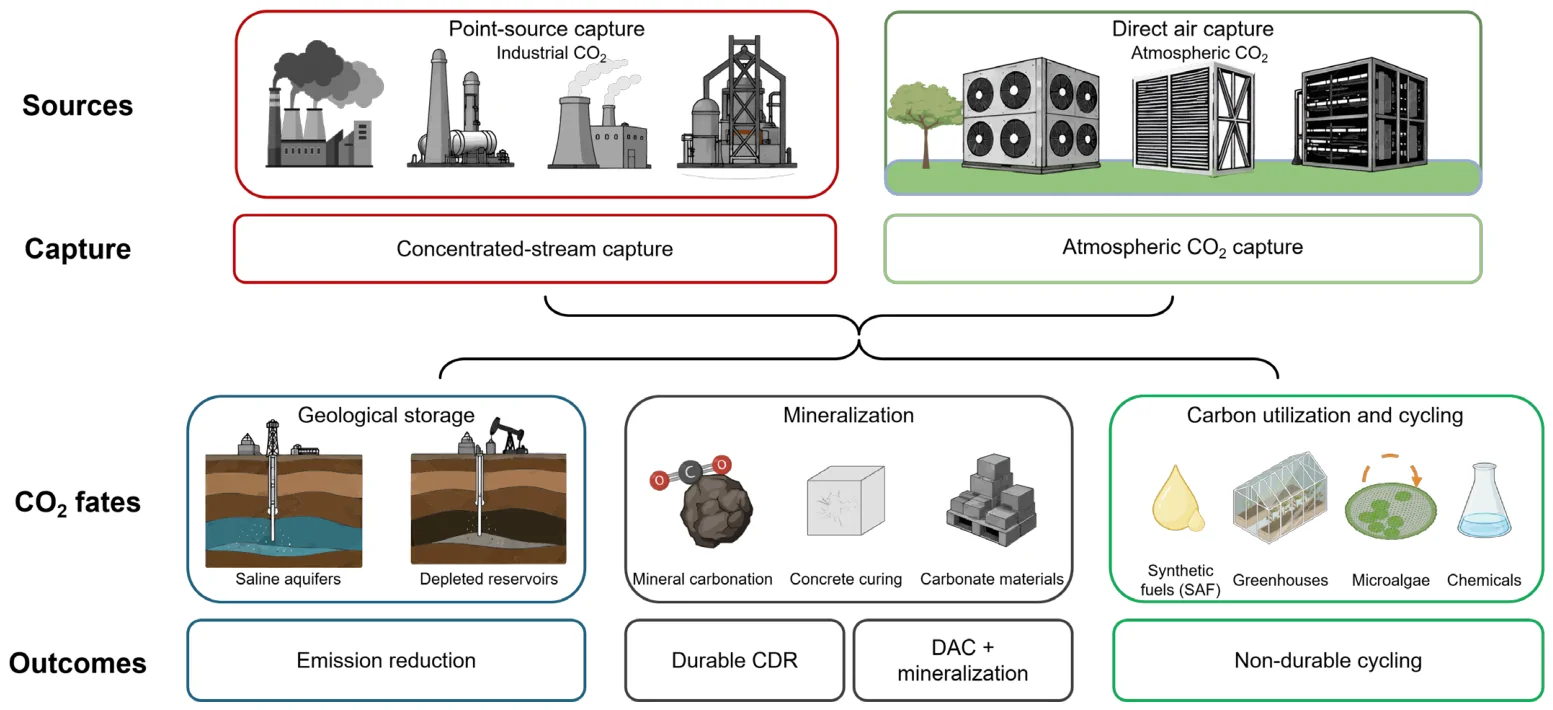
Positioning DAC within carbon-removal pathways and carbon fate.

Figure 1 positions DAC among carbon-removal pathways and distinguishes durable removal from carbon utilization and temporary cycling.

## 2. Capture Chemistry and Mechanisms

DAC requires a binding strength for CO_2_ that is sufficient to enable uptake from ambient air yet reversible enough to permit efficient regeneration. At a concentration of approximately 400 ppm, insufficient affinity yields low working capacity, while excessive affinity raises desorption energy, slows cycling, and may accelerate degradation [13]. Capture chemistries should therefore be compared not by equilibrium uptake alone but by their binding pathway, regeneration requirement, humidity response, and failure boundary. Such a comparison clarifies which sorbent structures, contactor geometries, and regeneration modes each mechanism can accommodate.

### 2.1. Liquid Alkaline Systems

Hydroxide and carbonate systems rank among the most mature DAC chemistries. They use strong alkaline media to convert atmospheric CO_2_ into carbonate or bicarbonate species, followed by regeneration of the alkaline solvent or sorbent. A typical liquid-alkali process couples air contact with solvent regeneration: KOH or NaOH absorbs CO_2_ to form carbonate, which then reacts with Ca(OH)_2_ to precipitate CaCO_3_ and regenerate the alkaline solution [29,30]. High-temperature calcination, typically at about 900 °C, decomposes CaCO_3_ into CaO and concentrated CO_2_, while hydration of CaO regenerates Ca(OH)_2_ and closes the cycle [31,32]. This route offers well-established chemistry, strong capture ability, and a high-purity CO_2_ product.

The limiting step, however, is not air absorption but carbonate regeneration [33]. Calcination transfers the separation burden to high-temperature heat, solids handling, oxygen supply, heat integration, and CO_2_ purification. These factors determine water balance, process emissions, and life-cycle removal efficiency. Alternative pH-swing routes can reduce part of the calcination penalty, but they introduce new losses through membrane resistance, ion transport, crystallization control, and stack scale-up [34]. Strong alkaline capture thus serves as a performance baseline rather than proof of DAC viability. The route becomes scalable only when alkali recovery, carbonate regeneration, water management, solids circulation, and CO_2_ purification are integrated without converting rapid air capture into a high-cost, high-emission process.

### 2.2. Solid Amine Chemisorption

Amine chemisorption is one of the most widely investigated trapping mechanisms in solid-sorbent DAC. Amino groups chemically interact with CO_2_ at low partial pressure to form ammonium carbamate, carbamic acid, or bicarbonate species [35]. Compared with physical sorbents that rely on weak van der Waals forces, amine-based materials offer higher affinity and selectivity at 400 ppm CO_2_, and they can be regenerated at relatively low temperatures, typically 80–120 °C, which facilitates coupling with industrial waste heat, geothermal energy, or heat pumps [36].

Under dry conditions, primary and secondary amines typically cooperate through two amine sites to capture one CO_2_ molecule, forming ammonium carbamate [37,38]. This mechanism provides strong binding but limits theoretical amine efficiency to 0.5 CO_2_ per nitrogen atom. In wet air, water participates in proton transfer, favoring bicarbonate formation, where ideally one amine site stabilizes one CO_2_ molecule [39]. The actual bicarbonate contribution depends on local water environment, amine type, pore structure, and proton migration pathways, and not all amine-based materials achieve efficient monoamine capture under humid conditions.

**Figure 2:**
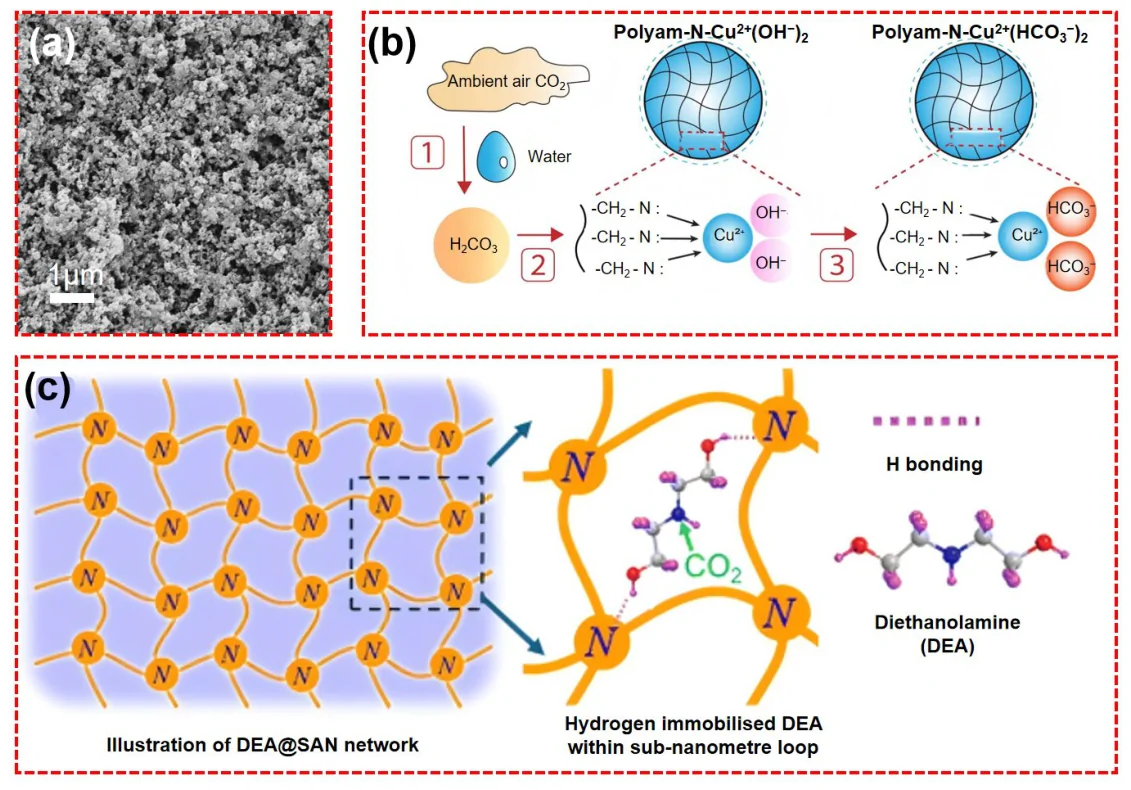
Representative sorbent architectures and water-mediated CO_2_-capture mechanisms under DAC-relevant conditions. (**a**) Cross-sectional SEM image showing macroporous resin architecture for gas diffusion. (**b**) Schematic mechanism of water-mediated CO_2_ uptake and bicarbonate stabilization in anion-exchange sorbents, where 1–3 denote CO_2_ hydration, reaction with Cu^2+^–OH sites, and bicarbonate formation, respectively. (**c**) Confined amine domains in polymer networks that improve amine accessibility and suppress pore blockage. Reproduced/adapted with permission from Refs. [41,42,43].

Amine-based sorbents encompass impregnated materials, grafted species, functionalized polymers, aminated metal–organic frameworks, and cellulose- or oxide-supported composites. Impregnated materials offer high amine loading and initial capacity but suffer from amine loss, volatilization, pore blockage, and insufficient cyclic stability. Grafted materials resist amine loss better, yet their amine loading and total capacity may be limited. Crystalline materials such as diamine-grafted MOFs provide well-defined structural environments, but their water stability, preparation cost, and large-scale shaping remain unproven. The key metric for amine DAC is not total amine content but the fraction of sites that remains accessible, reactive, and regenerable during wet-air cycling. High loading improves DAC only when it does not block pores, slow CO_2_ diffusion, increase water uptake, or accelerate oxidative and thermal degradation [40]. Representative sorbent architectures and water-assisted capture mechanisms are illustrated in Figure 2.

### 2.3. Electrochemical Capture and Emerging Routes

Beyond conventional heat-driven absorption and adsorption, electrochemical DAC has emerged as a significant frontier. This approach controls CO_2_ affinity through applied potential, redox state, local pH, or ion migration to drive capture and release [44]. Electrochemical processes can act directly on reactive sites or solution chemistry, avoiding the need to heat the entire sorbent bed or solvent inventory. This feature makes them attractive for coupling with renewable electricity and distributed modules.

Electrochemical DAC can be grouped into direct and indirect routes. Direct capture exploits reversible changes in CO_2_ affinity at redox-active sites; for example, reduced quinones form CO_2_ adducts and release CO_2_ upon oxidation. Indirect routes release CO_2_ and regenerate the capture medium by changing solution pH, regenerating hydroxide ions, shifting carbonate-bicarbonate equilibria, or separating acid and base streams via bipolar membrane electrodialysis [45]. Key challenges include oxygen side reactions, Faradaic inefficiency, membrane and electrode cost, coupled transport, and module scale-up [46].

Other emerging routes, such as ultramicroporous physisorption, facilitated-transport membranes, mineralization-assisted capture, and integrated capture-conversion systems, remain at earlier stages [47]. These mechanisms are mechanistically distinct from the chemical routes described above, and they should be selected together with their operating pathways. Redox sites, pH-swing systems, membranes, and hybrid routes may appear effective in small cells or batch tests, yet they lose value when oxygen crossover, water transport, membrane resistance, pressure drop, CO_2_ purity, and storage requirements are included [48,49]. Mechanism selection should therefore begin with the intended module, regeneration mode, energy input, and carbon destination. A summary of the major DAC capture chemistries, their regeneration methods, and the main system-level penalties is provided in Table 1.

## 3. Design Principle of DAC Sorbent

**Figure 3:**
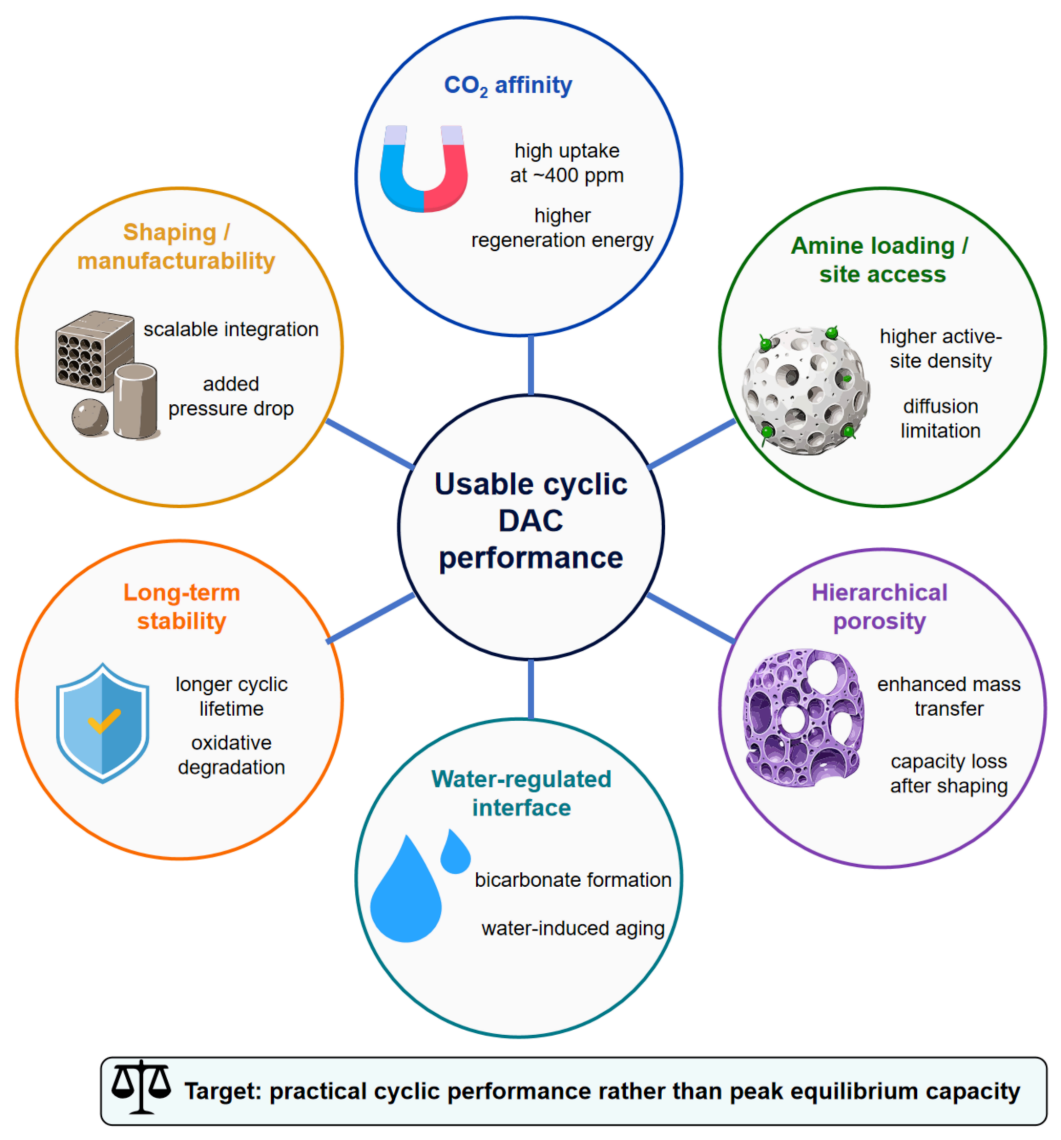
Key constraints governing practical DAC sorbent performance.

While the preceding chapter discussed DAC capture chemistries according to their reaction pathways and regeneration characteristics, this chapter focuses on how these mechanisms determine practical sorbent behavior under dilute CO_2_ concentration, humid air, and repeated cycling conditions. Unlike concentrated flue gas capture, DAC requires sorbents to maintain sufficient low-pressure affinity, rapid mass transfer, and long-term stability simultaneously [51,52]. Therefore, sorbent performance should not be evaluated only by initial adsorption capacity, but by the ability to preserve accessible active sites, effective transport pathways, regeneration compatibility, and structural integrity throughout operation. These coupled requirements are summarized in Figure 3.

### 3.1. CO_2_ Affinity and Regenerability

CO_2_ affinity is the basis for capturing molecules from ambient air, but its practical value depends on whether the captured CO_2_ can be released efficiently during regeneration. Weak interactions usually provide insufficient uptake at approximately 400 ppm CO_2_ [13]. Increasing the interaction strength through polar groups, alkaline sites, amines, open metal sites, ultramicropores, or redox-active centers can improve low-pressure capture, but these same interactions may increase desorption energy, strengthen water retention, and accelerate material degradation [53,54].

An effective DAC sorbent therefore requires a balanced affinity rather than the strongest possible binding. Excessively strong sites can improve adsorption under dilute conditions but may slow CO_2_ release and increase regeneration requirements. The suitable affinity range should enable rapid uptake at low partial pressure while maintaining reversible adsorption and desorption under thermal, vacuum, steam, or electrochemical regeneration conditions. This balance should be determined through cyclic adsorption–desorption performance rather than equilibrium uptake alone [55].

### 3.2. Accessibility of Active Sites and Amine Efficiency

Active-site accessibility determines whether the designed chemical functionality can contribute to practical CO_2_ capture capacity. High nitrogen content or increased amine loading does not necessarily lead to higher working capacity because some active sites may become buried, aggregated, diffusion-limited, blocked by water, or difficult to regenerate [56]. The more meaningful criterion is the fraction of active sites that can repeatedly participate in CO_2_ adsorption and release during realistic DAC cycles.

In amine-based sorbents, the relationship between amine loading and capture efficiency is controlled by the balance between active-site quantity and accessibility. Increasing amine content can provide more theoretical reaction sites, but excessive polymer accumulation may occupy pore space, restrict gas transport, and reduce regeneration efficiency. Therefore, sorbent design should focus on maintaining a high proportion of accessible and reactive amine sites rather than simply increasing nitrogen content.

Amine efficiency is commonly expressed by the CO_2_/N molar ratio, but this value only reflects practical performance when site accessibility and regeneration completeness are considered together. Under dry conditions, primary and secondary amines generally require two amine sites to stabilize one CO_2_ molecule [37,57]. In humid air, water-assisted bicarbonate formation may improve apparent efficiency by allowing one amine site to participate in CO_2_ capture, although the actual contribution depends on amine structure, water activity, site distribution, and proton-transfer pathways. The key objective is therefore to improve the repeated utilization of amine sites during cycling rather than maximizing total amine content [58].

### 3.3. Hierarchical Porosity and Diffusion Control

Pore architecture determines both the amount of CO_2_ captured and the rate at which CO_2_ molecules reach active sites. Because atmospheric CO_2_ concentration and molecular flux are extremely low, diffusion limitations become more significant in DAC than in many conventional adsorption processes. Materials that exhibit high capacity in static measurements may lose practical performance under airflow conditions because of extended diffusion paths, restricted channels, viscous functional phases, or slow intraparticle transport [59,60].

Effective DAC sorbents generally require coordinated pore structures in which different pore scales contribute to different transport functions. Micropores improve CO_2_ recognition and selectivity, mesopores facilitate functional-group distribution and molecular diffusion, while macropores promote bulk gas transport and reduce resistance within shaped sorbents [61,62,63]. However, excessive functionalization may reduce available pore volume and offset the benefit of increased porosity [64,65]. Therefore, pore design should be optimized according to dynamic uptake, pressure drop, mechanical strength, and performance after shaping, rather than pore volume alone [66].

### 3.4. Humidity Adaptability and Water-Control Interface

Humidity is a decisive factor in DAC sorbent performance because atmospheric operation inevitably involves fluctuating water vapor. Water can either promote CO_2_ capture by participating in reaction pathways or reduce performance by competing for adsorption sites, increasing regeneration energy, and damaging material structures. Consequently, water should be considered as an active component that regulates capture thermodynamics, kinetics, and cycling behavior rather than merely as an unwanted impurity.

In amine-based solid sorbents, water acts primarily as a reaction variable that shifts the thermodynamic equilibrium of CO_2_ binding. Moderate hydration can facilitate bicarbonate formation or hydrated intermediates, thereby improving CO_2_ uptake and transport kinetics [37,39]. However, excessive water raises the latent heat load during regeneration, occupies pore space, and may accelerate amine loss or framework hydrolysis [54]. Water-regulated DAC materials must therefore control local water activity near active sites without incurring a regeneration penalty.

Beyond amine systems, moisture-swing behavior in certain anion-exchange resins and quaternary ammonium salts illustrates how hydration state can be engineered as a regeneration trigger. In these materials, dry conditions favor CO_2_ adsorption as carbonate or bicarbonate species, whereas humidity exposure alters ion-pair stability and promotes release [67,68]. Whether water assists capture or drives regeneration, the material-level challenge remains the same: maintaining suitable local hydration to support proton transfer and bicarbonate formation while preventing excessive condensation, hydrothermal aging, and structural damage.

Water-related degradation depends strongly on material chemistry and operating conditions. In amine-grafted silicas, water can accelerate silica hydrolysis and amine leaching [69]. In MOFs, moisture may weaken metal–ligand coordination and compromise framework stability [70]. Polymer-supported amines may experience plasticization, which can improve chain mobility but also promote aggregation and oxidative degradation [40]. These effects are governed by temperature, humidity, cycle number, and support structure.

An ideal DAC material should regulate water participation rather than simply tolerate moisture exposure. Strategies including hydrophobic framework design, spatial control of hydrophilic sites, protective coatings, cross-linked amine networks, and water-stable porous structures have been explored [70,71]. Ultimately, humid cyclic performance, including capacity retention, transport behavior, and energy demand, provides a more reliable evaluation than dry-gas adsorption results alone [54].

### 3.5. Stability, Cost and Manufacturability

Long-term DAC deployment requires sorbents to maintain performance under oxygen exposure, humidity variation, repeated regeneration, and large-scale cycling conditions. Initial adsorption capacity alone cannot determine practical value because degradation during operation may reduce lifetime performance. Solid amine materials can suffer from thermal degradation, oxidation, amine volatilization, urea formation, amidation, support hydrolysis, and pore collapse [69,72]. Therefore, resistance to oxidative and hydrothermal aging is often more representative of application potential than fresh material capacity measured under ideal conditions.

Cost and manufacturability ultimately determine whether advanced sorbents can move beyond laboratory studies. Many high-performance materials depend on expensive precursors, complex synthesis procedures, organic solvents, or strict activation processes, which may limit large-scale production. Future DAC sorbents must integrate adsorption performance with affordable raw materials, scalable manufacturing, structural formability, mechanical durability, and low environmental impact [27,73]. Material development should also consider shaping, coating, pressure drop, particle wear, and replacement frequency at an early stage rather than treating these factors as later engineering adjustments [74,75,76]. Practical sorbent value is therefore determined by sustained working capacity over the entire service lifetime. The key trade-offs in DAC sorbent design, including affinity, loading, porosity, water management, shaping, and stability, are summarized in Table 2.

## 4. Structured Contactors and Regeneration Engineering

While the preceding chapter established DAC sorbent design principles at the material level, this chapter examines how these properties are maintained when sorbents are transformed from laboratory powders into practical contactors and how regeneration strategies influence the overall efficiency of CO_2_ recovery. Because DAC requires processing extremely large volumes of air, practical systems are strongly affected by transport resistance, pressure drop, heat transfer behavior, and regeneration duration [26]. Therefore, the transition from material performance to system performance cannot rely solely on adsorption capacity measured under ideal conditions. Instead, the losses introduced during shaping, airflow management, heat and mass transfer, and regeneration must be considered together, because these factors determine whether intrinsic sorbent properties can be converted into stable cyclic CO_2_ removal performance [79].

### 4.1. From Powder to Structured Forms: Addressing Shaping Loss

Most DAC sorbents are initially developed and evaluated in powder form because powders provide convenient characterization conditions, short diffusion distances, and high exposure of active sites, making them suitable for understanding adsorption mechanisms and screening new materials [50]. However, these advantages do not directly translate into large-scale operation. When powders are applied in practical contactors, they often generate excessive pressure drop, increase fan energy consumption, and suffer from problems associated with particle aggregation, channel formation, dust generation, and limited heat transfer. These effects become increasingly significant during regeneration because poor thermal transport can create uneven temperature distributions, resulting in incomplete CO_2_ release or additional energy consumption [26,28].

**Figure 4:**
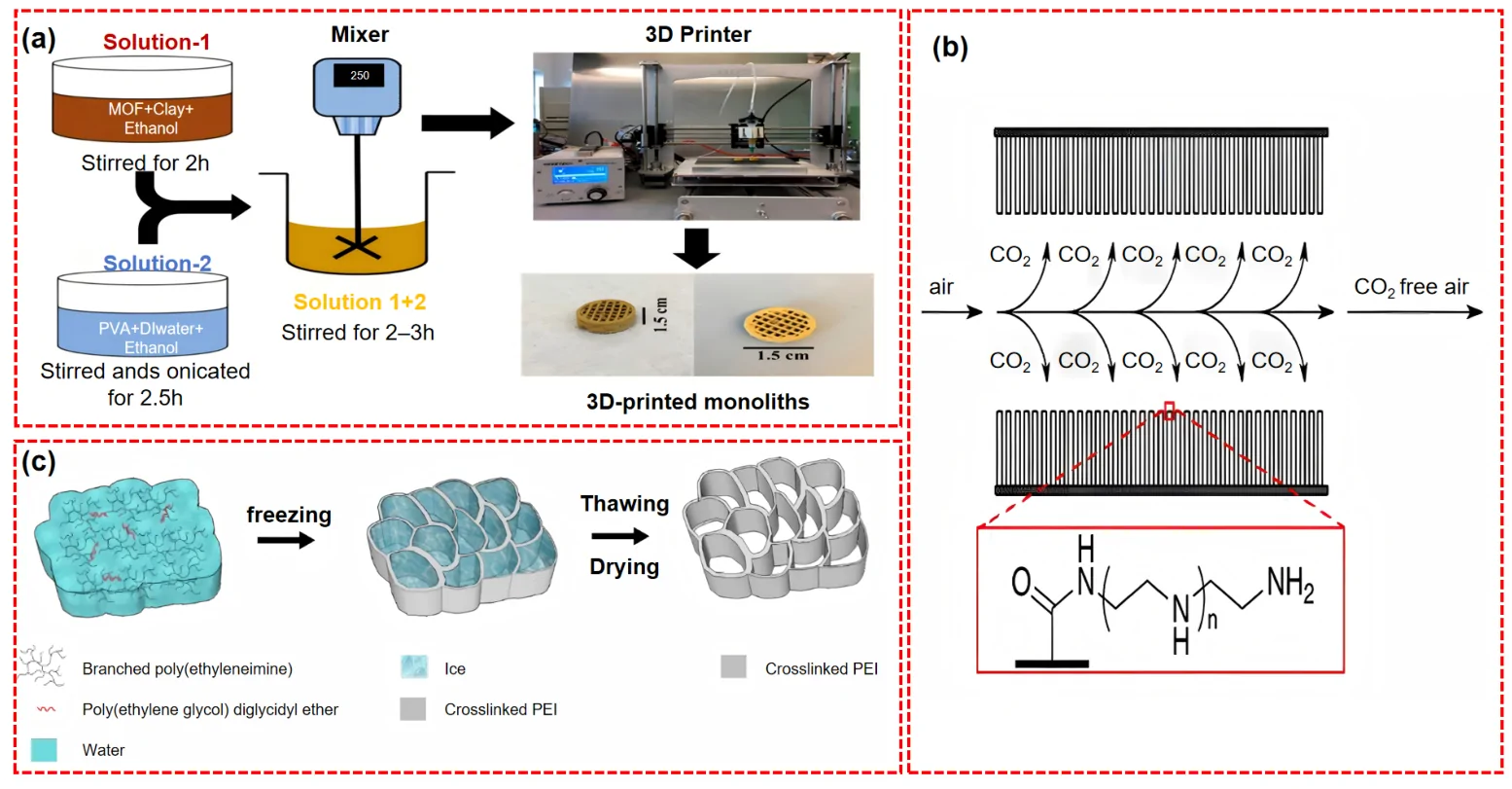
Representative structured sorbents for DAC applications. (**a**) 3D-printed MOF monoliths as geometrically controlled gas adsorbents with tunable channels and shortened transport pathways. (**b**) Amine-grafted hollow fiber adsorbent designed to enhance gas contact and reduce intraparticle diffusion resistance. (**c**) Self-supporting PEI-based adsorbent that avoids loose-powder operation and improves contactor compatibility. Reproduced/adapted with permission from Refs. [80,81,82].

To overcome these limitations, powder sorbents must be converted into structured forms that provide sufficient gas accessibility while maintaining active-site utilization. Approaches such as granulation, extrusion, coating, fiber formation, monolithic structures, and additive manufacturing have therefore been explored for DAC applications [74,80]. Representative structured sorbent architectures are shown in Figure 4.

The purpose of structuring is not simply to preserve the adsorption capacity obtained from powder materials, but to improve the overall utilization of active sites under realistic operating conditions. Particle-based structures provide relatively simple manufacturing routes and good packing characteristics, but they may introduce diffusion limitations and reduce transport efficiency. Monolithic and honeycomb structures can decrease pressure drop and improve airflow distribution, although their performance depends strongly on coating thickness, active material loading, and mechanical stability during repeated cycling [83,84,85,86]. Fiber-based architectures shorten diffusion distances and increase gas contact efficiency, but their preparation complexity and module integration requirements remain important considerations [87,88,89]. Three-dimensional printing provides additional control over channel geometry and structural design, offering opportunities to optimize mass transfer and mechanical performance simultaneously [90,91].

The shaping process inevitably changes the relationship between material properties and practical operation. Active-site dilution, pore blockage, increased diffusion resistance, additional thermal mass, coating detachment, mechanical wear, and pressure-drop growth may all reduce the benefit expected from the original powder sorbent [74,83]. Therefore, structured sorbents should be evaluated according to their retained performance after shaping, including breakthrough behavior, cyclic capacity, regeneration efficiency, and volumetric productivity, rather than relying only on the adsorption capacity of unstructured materials.

### 4.2. Contactor Geometry and Pressure Drop Control: Addressing Flow Loss

The air contactor determines how effectively atmospheric CO_2_ reaches the sorbent and directly affects fan power demand, equipment scale, capture productivity, and overall system cost. Unlike point-source capture, DAC processes air with extremely low CO_2_ concentration and therefore requires much larger gas throughput to obtain equivalent carbon removal rates [92,93]. Under these conditions, even moderate pressure losses can significantly increase electricity consumption because the energy required for air movement becomes a major component of system operation.

Contactor design must therefore be developed together with sorbent characteristics rather than treated as an independent engineering step. Increasing gas–solid contact area can improve CO_2_ transport and reduce dependence on extremely high sorbent affinity, but excessive contact area may increase flow resistance and complicate equipment design [94,95]. Fixed-bed systems provide simple operation and mature engineering experience, yet their application in DAC is limited by pressure drop, internal diffusion resistance, and heat-transfer constraints [96]. Alternative configurations improve transport behavior by modifying gas pathways and material arrangement. Radial-flow contactors reduce flow distance and pressure loss, while moving-bed and circulating systems allow continuous operation through separation of adsorption and regeneration zones, although they introduce additional challenges related to mechanical stability, sorbent circulation, and equipment complexity [28,97,98].

Monolithic and honeycomb contactors are particularly attractive for DAC because their parallel channels provide low resistance to airflow and enable efficient gas contact through sorbent coatings deposited on channel surfaces [78,83]. However, increasing coating thickness does not necessarily improve practical performance. Excessive loading may increase nominal CO_2_ capacity while reducing active-site accessibility, extending diffusion pathways, increasing thermal demand, and slowing regeneration [99,100]. Therefore, contactor optimization should focus on the amount of CO_2_ captured per unit volume and per unit energy input rather than maximizing sorbent loading alone.

### 4.3. Coupled Heat and Mass Transfer: Addressing Thermal and Transport Loss

The DAC contactor should be regarded as a coupled heat and mass transfer system rather than simply a container for sorbent materials. During adsorption, CO_2_ molecules must first move through the bulk airflow, pass across the gas boundary layer, enter the porous structure, and finally interact with active sites through diffusion and reaction processes. These transport steps are strongly influenced by external mass transfer, intraparticle diffusion, polymer-phase mobility, pore accessibility, and reaction kinetics, all of which determine breakthrough behavior and working capacity under practical operating conditions [101]. Because atmospheric CO_2_ concentration is extremely low, even moderate transport resistance can substantially reduce effective capture rates and limit the utilization of available active sites [102].

The interaction between heat generation and mass transfer further complicates DAC operation. CO_2_ adsorption on amine groups or strongly basic sites is generally accompanied by heat release, which can increase local temperature and alter adsorption equilibrium, water distribution, polymer mobility, and CO_2_ diffusion behavior [56,103]. In large-scale contactors, uneven heat distribution may create temperature gradients that affect adsorption efficiency and cycle stability. During regeneration, insufficient heat transfer can prevent complete CO_2_ desorption, whereas excessive local heating may accelerate sorbent degradation and reduce long-term durability [56,104].

Water migration introduces an additional coupling between thermal behavior, mass transport, and material stability. Under humid air conditions, water does not only compete with CO_2_ for adsorption sites but also participates in reaction pathways and influences heat consumption during regeneration [103]. Therefore, breakthrough profiles, temperature evolution, and moisture transport should be analyzed together because these parameters collectively determine where CO_2_ adsorption occurs, how efficiently heat is transferred, and whether regeneration can be completed within a practical cycle time. Improving DAC contactor performance requires simultaneous optimization of gas transport, thermal management, and moisture control rather than focusing on a single transport parameter.

### 4.4. Regeneration Methods and Energy Matching: Addressing Regeneration Loss

Regeneration determines whether captured CO_2_ can be recovered efficiently while maintaining the energy advantages obtained during adsorption. In solid-sorbent DAC systems, CO_2_ is first concentrated from atmospheric air and subsequently released by modifying temperature, pressure, purge conditions, or electrochemical states [102]. However, regeneration energy is not consumed exclusively by CO_2_ desorption. Additional energy losses arise from heating inactive support materials, contactor structures, co-adsorbed water, and surrounding components, which reduces the fraction of supplied energy directly contributing to CO_2_ recovery [78,105]. Therefore, regeneration strategies should be evaluated according to recovered CO_2_ per unit energy input, product quality, cycle duration, and impact on sorbent lifetime rather than regeneration temperature alone [102].

Temperature-swing adsorption (TSA) remains widely used because it can utilize relatively low-temperature heat sources, but its efficiency depends on how effectively thermal input is transferred to CO_2_ desorption. A considerable portion of supplied heat may be consumed by the sorbent matrix, structural materials, and retained water rather than directly promoting CO_2_ release [106,107]. Vacuum-swing adsorption (VSA) enhances desorption by reducing CO_2_ partial pressure, but deeper vacuum conditions increase electrical demand, sealing requirements, and equipment complexity. Temperature–vacuum swing adsorption (TVSA) combines thermal and pressure effects to improve regeneration performance, although it introduces additional coordination requirements involving heating, evacuation, cooling, and subsequent adsorption recovery [108]. Steam-assisted regeneration can provide both heat and purge effects, but it may increase water-related penalties, including condensation management, product drying requirements, hydrothermal aging, and potential amine loss [109].

Electrified regeneration provides another pathway for reducing dependence on external heat sources, but lower-temperature or electricity-driven operation does not automatically guarantee lower net energy consumption. Solid amine sorbents generally require less regeneration temperature than liquid alkaline systems, yet total cycle energy is also influenced by heat capacity, water uptake, cooling requirements, CO_2_ purity, and regeneration completeness [43]. Electrochemical regeneration avoids heating the entire sorbent bed or solvent inventory, but practical performance remains dependent on factors such as oxygen interference, Faradaic efficiency, electrode stability, membrane resistance, crossover effects, and module durability. Electrification is beneficial only when localized energy input reduces overall energy consumption while maintaining CO_2_ purity, sorbent lifetime, and system productivity [110].

A meaningful comparison between regeneration approaches therefore requires consistent evaluation boundaries. Energy consumption should be considered together with product concentration, cycle productivity, water management, heat recovery potential, and degradation behavior [111,112]. The optimal regeneration method is not determined by the lowest operating temperature or the simplest energy input mode, but by whether it enables efficient, durable, and scalable conversion of captured CO_2_ into a usable product stream.

## 5. System Integration, Economics, and Scalable Deployment

While the preceding chapter examined how sorbent performance can be transferred from materials to structured contactors through shaping and regeneration engineering, this chapter evaluates whether captured CO_2_ can ultimately contribute to net and durable carbon removal after considering the complete process chain. A DAC system does not end at the point of CO_2_ capture, because energy supply, sorbent replacement, product conditioning, compression, transport, and storage determine whether atmospheric carbon is actually removed. Losses occurring at any stage may reduce the climate benefit obtained from high device-level capture performance. Therefore, DAC should be evaluated through the relationship among gross CO_2_ captured, net CO_2_ removed, and durable CO_2_ stored rather than through capture capacity alone. Table 3 provides a comparative overview of the major DAC pathways, summarizing their primary advantages, the dominant performance losses encountered during scale transition, and the most appropriate evaluation metrics for each route.

### 5.1. Main Process Architecture

The DAC technologies closest to commercialization are primarily based on liquid-solvent and solid-sorbent configurations. Liquid-solvent DAC generally relies on strong alkaline solutions to absorb atmospheric CO_2_, after which the captured carbon is recovered through a series of chemical regeneration processes. A representative pathway couples KOH absorption with carbonate formation, precipitation, calcination, and alkaline recovery [123,124]. This approach benefits from mature reaction chemistry, strong absorption capability, and the ability to produce a concentrated CO_2_ stream suitable for downstream handling. However, the overall performance depends strongly on the integration of high-temperature heat supply, oxygen management, thermal recovery, and large-scale process equipment, which together influence both economic cost and life-cycle emissions.

Solid-sorbent DAC follows a more modular process configuration. Air is driven through a contactor where CO_2_ is captured at ambient or moderately elevated temperatures, and the saturated sorbent is subsequently regenerated through changes in temperature, pressure, humidity, or combined operating conditions. The possibility of using lower-temperature energy sources provides advantages for distributed deployment and integration with renewable or waste energy systems. Nevertheless, practical performance remains dependent on maintaining sorbent lifetime, controlling pressure drop, limiting water-related penalties, and reducing the energy demand associated with vacuum operation and module operation [78].

The comparison between liquid and solid DAC systems should therefore focus on process compatibility rather than individual capture capacity. Liquid systems may be advantageous where centralized facilities can integrate large-scale heat supply, chemical recovery, and storage infrastructure. Solid systems provide greater flexibility for modular deployment but require improvements in sorbent durability, contactor design, and regeneration efficiency. The preferred architecture ultimately depends on the interaction between local energy availability, water conditions, storage accessibility, and the requirements of downstream CO_2_ management.

**Figure 5:**
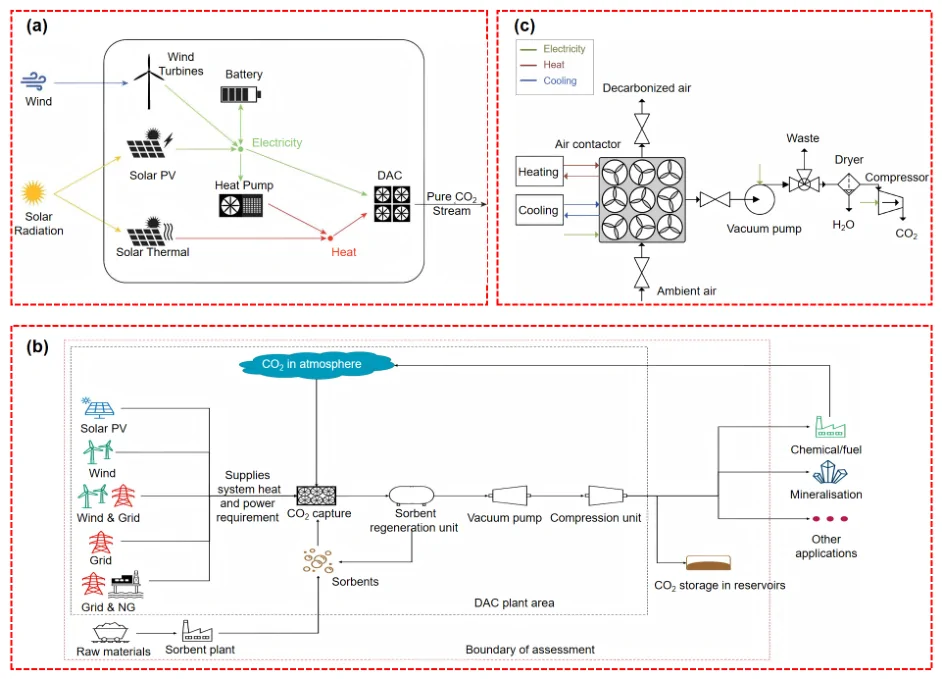
Process architectures and system integration pathways for DAC. (**a**) Integration of DAC into a multi-energy system supplied by low-carbon solar or wind resources. (**b**) Simplified DAC flow diagram defining the process boundary from air contact and capture to regeneration, CO_2_ conditioning and downstream handling. (**c**) Solid-sorbent DAC flowsheet showing adsorption, regeneration and CO_2_ recovery steps. Reproduced/adapted with permission from Refs. [104,125,126].

Figure 5 illustrates the process integration pathways, including DAC coupled with multi-energy systems, a simplified process boundary description, and the cyclic adsorption–desorption operation of modular solid-sorbent DAC [104,125,126].

### 5.2. Emerging DAC Architectures and Carbon-Utilization Boundaries

Emerging DAC configurations should be evaluated according to their position within the carbon-removal chain rather than being compared only by capture mechanism. Electrochemical DAC remains part of engineered carbon removal when it produces a concentrated CO_2_ stream that can be directed toward durable storage or verified long-lived applications. Its practical value depends on the coordinated performance of the entire electrochemical system, because improvements in capture chemistry alone cannot compensate for losses caused by inefficient charge transfer, material instability, or insufficient product concentration.

Passive and semi-passive DAC approaches reduce the requirement for active air movement by using naturally available airflow or environmental gradients. However, reduced fan energy does not necessarily translate into lower overall removal cost. Lower airflow control and weaker transport conditions may require larger sorbent quantities, extended operating periods, or increased land requirements to achieve comparable carbon removal rates. Therefore, passive approaches are more suitable for specific conditions where environmental advantages compensate for reduced process control rather than serving as direct replacements for engineered contactors [127].

Hybrid DAC systems require particular attention to carbon destination because the same capture process can lead to different climate outcomes depending on subsequent handling of the recovered CO_2_. Mineralization-based pathways can contribute to durable carbon removal when carbonate stability, material supply, energy consumption, and monitoring requirements are properly addressed [128,129]. In contrast, pathways that convert captured CO_2_ into fuels or short-lived products mainly function as carbon utilization because the carbon is likely to return to the atmosphere within a limited time period [130]. Biological fixation approaches occupy an intermediate position because their climate benefit depends on whether the resulting carbon-containing products can achieve sufficiently long storage duration and reliable verification [131].

### 5.3. Techno-Economic Drivers

DAC cost estimates are highly sensitive to the chosen process boundary, and a low reported figure often results from excluding essential items such as compression, storage, sorbent replacement, water management, land use, or financing [28]. Any meaningful comparison across different DAC routes must therefore clearly state which cost components are included and which are omitted. For solid-sorbent systems, a high working capacity can reduce the required sorbent inventory only when the cycle time remains short, the pressure drop stays low, and regeneration is fully achieved. A material that exhibits high capacity but suffers from slow breakthrough, demands deep vacuum, or degrades rapidly will actually increase overall cost through larger contactors, longer cycle times, and more frequent replacement. For liquid-solvent DAC, the cost is dominated less by the absorption chemistry itself and more by the supporting unit operations, including air contactors, causticization, solid–liquid separation, calcination, oxygen supply, heat recovery, CO_2_ compression, and water balance. Energy costs also differ by type, with electricity powering fans, pumps, vacuum systems, and compression, while heat is required for regeneration. A techno-economic analysis should therefore identify the limiting cost driver for each specific architecture rather than relying on a single average value to compare different routes.

### 5.4. Key Barriers to Scalable Deployment

Reaching Gt-scale DAC deployment requires overcoming four interconnected barriers that involve sorbent supply chains, module manufacturing, site constraints, and the matching of storage capacity with monitoring and verification. Each of these areas demands progress in parallel with material and process development. Many high-performance sorbents, particularly metal–organic frameworks, functionalized polymers, and advanced amine composites, depend on expensive ligands, organic solvents, or multi-step synthesis that remain viable only at gram scale but become economically prohibitive when production is scaled to tons [27]. Even relatively inexpensive amine-impregnated materials face supply-chain limitations in the availability of polyethyleneimine and silica supports, as well as challenges in precursor purity and consistency. The transition from laboratory synthesis to continuous industrial production introduces additional difficulties in quality control, batch-to-batch reproducibility, and solvent recovery, all of which must be solved before sorbents can be procured at the scale required for million-ton-per-year DAC plants. Solid-sorbent DAC offers a potential path to cost reduction through modular fabrication and factory replication, but realizing this potential requires standardized module designs, automated assembly lines, and quality assurance protocols that maintain performance across thousands of identical units [78]. Liquid-solvent DAC, by contrast, relies on centralized, custom-engineered facilities that benefit from economies of scale but face long construction times, high upfront capital, and site-specific engineering. The trade-off between modular replication and centralized scale remains unresolved and will likely depend on local energy, land, and storage conditions. Site selection is also constrained by substantial land area requirements for air contactors, especially for solid systems with lower capture rates per unit ground area, and by water consumption, primarily through evaporative loss in liquid systems and humidity management in solid systems [9,58]. These constraints become particularly acute in arid regions where low-carbon energy is abundant but water is scarce [16,17]. Climate variability further complicates deployment because temperature and humidity fluctuations affect sorbent capacity, cycle time, and regeneration energy, requiring either adaptive operation or overdesign that raises capital cost. Finally, the primary durable sinks for DAC, namely geological storage and mineralization, face their own scale-up challenges: storage site exploration, characterization, and permitting can take years to decades, and injection well capacity limits the rate of CO_2_ disposal. The spatial mismatch between favorable DAC deployment regions and available storage reservoirs adds transport cost and infrastructure requirements. Monitoring, reporting, and verification frameworks, which are essential for confirming permanence and enabling carbon crediting, must scale from pilot projects to industrial deployment without prohibitive cost increases [7,131]. These four barriers must therefore be tackled simultaneously if DAC is to contribute meaningfully to large-scale carbon removal.

### 5.5. Life Cycle Assessment and Net Carbon Removal

The goal of DAC is to remove CO_2_ from the atmosphere, but capture alone does not guarantee net-negative emissions. DAC systems generate emissions through construction, material production, sorbent replacement, energy supply, operation, maintenance, CO_2_ compression, transport, and storage. If the total life-cycle emissions approach or exceed the amount of CO_2_ captured, the climate benefit is greatly diminished [123,132]. The energy supply is a key determinant of overall performance: using high-carbon electricity or fossil-derived heat can substantially offset the capture benefit. Low-temperature solid-sorbent DAC may perform favorably in life-cycle assessment when driven by waste heat, geothermal energy, or low-carbon electricity, yet its net removal still depends on sorbent production, contactor manufacturing, module lifetime, water use, and replacement frequency. Life-cycle assessment must clearly distinguish between emission reduction, carbon cycling, and genuine negative emissions. DAC-derived fuels, greenhouse enrichment, biological fixation, indoor air management, and short-lived chemicals can reduce fossil carbon demand or provide local services, but they should not be counted as durable removal without verified long-lived storage. It is therefore essential to report gross CO_2_ captured, net CO_2_ removed, and durable CO_2_ stored as separate quantities so that device-level capture is not overstated as climate benefit [133].

### 5.6. Carbon Destination, Utilization, and Permanence

The ultimate climate value of DAC hinges on the fate of the captured CO_2_, which determines whether the process functions as durable removal, temporary storage, carbon utilization, or local air-quality management. Geological storage provides durable removal when injection, trapping, monitoring, and leakage control are properly verified. Mineralization offers a stable carbonate sink, though its scale depends on reaction kinetics, the availability of alkaline feedstocks, comminution energy, solid transport, and verification of carbonate permanence [128,129]. Long-lived products may delay re-emission only when the storage duration and substitution benefit are clearly demonstrated. In contrast, pathways such as fuel synthesis, greenhouse supply, short-lived chemicals, most biological products, and indoor air management mainly recycle carbon or provide local services and should not be assigned the same climate value. These different destinations should be ranked by storage duration, re-emission risk, substitution effect, life-cycle emissions, and the feasibility of monitoring and verification. Table 4 summarizes the main DAC architectures and their corresponding carbon-removal pathways.

## 6. Conclusions and Outlook

DAC research has already demonstrated that capturing CO_2_ from air is technically feasible, and the remaining challenge lies in retaining that capture performance through each subsequent step of the operational chain, including contactor operation, regeneration, product conditioning, and durable storage. This review has shown that performance is lost when molecular uptake fails to survive cycling, shaping, regeneration, product purification, or carbon storage. The performance-transfer framework developed here provides a systematic account of these cross-scale losses and identifies the dominant bottlenecks that determine whether laboratory-scale advances can translate into verifiable carbon removal, thereby distinguishing this review from previous surveys that have focused primarily on material properties or individual process stages. Future DAC research should therefore shift its primary focus from demonstrating capture feasibility to proving net removal under realistic and integrated conditions.

In the near term, the community should establish consistent reporting standards and validation protocols that move beyond simple indicators of material performance. Studies that report only static uptake, dry-gas capacity, or fresh-powder performance should no longer be accepted as sufficient evidence for DAC relevance. A minimum dataset for any new material or process should include low-pressure uptake, cyclic working capacity, breakthrough behavior, pressure drop after shaping, regeneration energy requirements, CO_2_ purity, water uptake, capacity retention over multiple cycles, and clearly defined test boundary conditions. These measurements should be conducted under specified humidity, temperature, flow, and regeneration conditions that reflect realistic operating environments. Although this step is primarily methodological, it is essential because techno-economic analysis and life-cycle assessment remain unreliable when critical parameters such as lifetime, pressure drop, product purity, and regeneration penalty are assumed rather than measured.

In the medium term, research efforts should integrate sorbent design with structured contactor development and regeneration protocol optimization. Material discovery should shift its objective from maximizing fresh uptake to preserving accessible active sites, maintaining diffusion pathways, and ensuring regenerability after shaping and aging. Contactors should be designed together with the sorbent rather than after powder optimization is complete, and regeneration conditions should be selected alongside contactor thermal mass, water loading, target CO_2_ purity, and downstream compression requirements. The most valuable module designs will be those that reduce pressure drop, shorten diffusion length, control water activity around active sites, and localize heat delivery without accelerating degradation.

Future DAC research should be prioritized according to the factors that most strongly determine practical carbon removal rather than solely pursuing higher adsorption capacity. At the material level, improving cyclic stability under realistic humidity and oxidative conditions should receive higher priority because performance degradation directly reduces lifetime carbon removal. At the engineering level, sorbent development should be coupled with contactor design and regeneration optimization to minimize losses introduced during shaping, airflow management and energy recovery. At the system level, standardized benchmarking protocols that integrate energy consumption, carbon intensity, water demand, cost and storage durability are required to enable meaningful comparison among different DAC pathways. These priorities indicate that future progress will depend less on maximizing isolated material properties and more on improving the transfer efficiency from molecular capture to verified net carbon removal.

In the long term, DAC deployment must be connected to low-carbon energy supply, CO_2_ transport infrastructure, durable storage sites, and monitoring and verification systems. A DAC plant delivers genuine negative emissions only when the captured CO_2_ remains net-negative after accounting for energy use, material replacement, compression, transport, and storage. Geological storage and mineralization require site characterization, continuous monitoring, leakage control, and robust accounting standards. Utilization pathways, including fuels, greenhouse enrichment, indoor air management, and most biological products, should be treated as carbon cycling or local service unless long-lived storage is verified. Deployment therefore depends not only on better materials and contactors but also on storage access, low-carbon heat and power, certification protocols, and transparent carbon accounting.

Future DAC research priorities should be determined by the factors that control the transfer of laboratory-scale performance into verified carbon removal rather than by isolated improvements in material properties. The highest priority should therefore be assigned to studies that evaluate DAC under realistic operating conditions, where cyclic performance, humidity effects, oxidative stability, structured-sorbent behavior, contactor-level pressure losses, and regeneration energy are considered together. These factors directly determine whether high intrinsic capture performance can be maintained after scale-up from materials to practical modules. In parallel, research should increasingly address product CO_2_ quality, water management, degradation mechanisms, and scalable manufacturing because these parameters influence operational reliability, environmental impacts, and deployment feasibility. Lower priority should be given to studies that report only equilibrium adsorption capacity under idealized conditions without considering cyclic operation, realistic gas composition, shaping effects, or complete energy accounting. Such studies remain useful for mechanism understanding, but they provide limited evidence for predicting practical DAC performance. Similarly, CO_2_ utilization pathways should be evaluated according to their carbon retention time and life-cycle impact, because utilization alone does not necessarily represent durable carbon removal. The remaining major challenge is the demonstration of integrated DAC systems at sufficiently large scale with verified net carbon removal. Although several DAC projects are progressing toward large-scale deployment, current operational experience remains limited compared with the scale required for climate mitigation scenarios. Future progress therefore depends not only on advancing capture materials, but also on integrating energy supply, regeneration, carbon transport, storage monitoring, and life-cycle verification into complete DAC systems.

## Figures and Tables

**Table 1:** Major DAC Capture Chemistries and System Penalties.

Route	Capture Mechanism	Regeneration	Main Advantage	Main Penalty	Key References
Hydroxide/carbonate	Alkaline conversion to carbonate/bicarbonate	Calcination or pH swing	Strong absorption, mature chemistry	High-temperature heat, caustic recovery	[16]
Solid amine	Carbamate (dry, 2 N per CO_2_) or bicarbonate (wet, 1 N per CO_2_)	TSA, VSA, TVSA or steam	High affinity at ~400 ppm CO_2_	Water uptake, degradation, pressure drop	[1]
Moisture swing	CO_2_ affinity changes with hydration state	Dry/wet cycling	Lower direct heat demand	Water demand, slow cycling, low CO_2_ purity	[50]
Electrochemical DAC	Redox, pH or ion-transport control	Electrical regeneration	Electrified operation	Voltage loss, crossover, electrode aging	[20]
Physisorption	CO_2_ confinement in micropores or polar sites	TSA or VSA	Fast, chemically simple	Weak uptake at ~400 ppm, H_2_O competition	[26]

**Table 2:** Key Trade-Offs in DAC Sorbent Design.

Design Factor	Benefit	Hidden Penalty	Better Evaluation Metric	Key References
Strong CO_2_ affinity	Higher uptake at ~400 ppm	Higher desorption energy, slower release	Cyclic working capacity	[55]
High amine loading	More nominal active sites	Pore blocking, diffusion limits	CO_2_/N efficiency, breakthrough capacity	[77]
Hierarchical porosity	Better access and transport	Capacity loss after functionalization/shaping	Dynamic uptake after shaping	[66]
Water-regulated interface	Promotes bicarbonate in some systems	Heat duty, hydrothermal aging	Humid cyclic performance	[54]
Shaping/coating	Enables contactor integration	Active-site dilution, pressure drop, thermal mass	Volumetric productivity and pressure drop	[78]
Long-term stability	Maintains lifetime performance	Oxidation, amine loss, framework aging	Capacity retention under wet-air cycling	[40]

**Table 3:** Comparative evaluation of major DAC technology pathways from material to system level.

DAC Route	Main Advantage	Dominant Performance Loss	Critical Evaluation Metric	Ref.
Liquid alkaline	Mature chemistry and high CO_2_ concentration	High-temperature regeneration and water management	Net removal energy and carbon intensity	[16,113,114]
Solid amine	Low-temperature regeneration and modularity	Oxidative aging, humidity effects and shaping loss	Cyclic working capacity and lifetime stability	[115,116,117]
Moisture swing	Reduced direct heat requirement	Water management and cycling rate limitations	CO_2_ release efficiency per water input	[118,119,120]
Electrochemical DAC	Direct electrical control of regeneration	Voltage loss, membrane resistance and electrode degradation	Electricity demand and long-term stability	[49,121,122]

**Table 4:** DAC Architectures and Carbon-Removal Pathways.

Pathway	Main Input	Product/Fate	Main Bottleneck	Removal Durability	Key References
Liquid DAC + storage	High-temperature heat, electricity, water	Concentrated CO_2_ for storage	Calcination, caustic recovery, compression	High if stored geologically	[123]
Solid DAC + storage	Low-grade heat, vacuum, electricity	CO_2_ for compression/storage	Sorbent lifetime, pressure drop, water uptake	High if stored durably	[133]
Electrochemical DAC	Low-carbon electricity	Variable CO_2_ purity	Cell resistance, crossover, stability	High only with durable storage	[134]
Passive/semi-passive DAC	Natural airflow or humidity gradient	Often dilute CO_2_ stream	Low throughput, large area, weak control	Uncertain	[127]
DAC + mineralization	Alkaline feedstock, grinding, transport	Stable carbonate	Feedstock supply, kinetics, MRV	High if carbonate permanence verified	[131]
DAC-to-fuels/short-lived use	CO_2_, H_2_/electricity, conversion unit	Fuel or chemical product	High energy use, re-emission	Usually non-durable	[130]

## Data Availability

Data sharing is not applicable to this article as no new data were created or analyzed in this study.
